# The Coffee–Acrylamide Apparent Paradox: An Example of Why the Health Impact of a Specific Compound in a Complex Mixture Should Not Be Evaluated in Isolation

**DOI:** 10.3390/nu12103141

**Published:** 2020-10-14

**Authors:** Astrid Nehlig, Rodrigo A. Cunha

**Affiliations:** 1 INSERM U 1129, Pediatric Neurology, Necker-Enfants Malades Hospital, University of Paris Descartes, 75015 Paris, France; nehliga@unistra.fr; 2 Faculty of Medicine, INSERM U 1129, 67000 Strasbourg, France; 3 CNC-Center for Neurosciences and Cell Biology, University of Coimbra, 3004-504 Coimbra, Portugal; 4 Faculty of Medicine, University of Coimbra, 3004-504 Coimbra, Portugal

**Keywords:** coffee, acrylamide, cancer, contaminant, exposure, consumption

## Abstract

The health implications of acrylamide in food are a matter of concern based on toxicological studies in rodents, which showed that doses of acrylamide more than 100 times higher than those estimated to result from dietary exposure in humans are carcinogenic; however, the cancer types reported in rodents are species-specific, and whether these results can be extrapolated to humans is still in question. In fact, human epidemiological studies revealed a general lack of association between dietary acrylamide exposure and the incidence of different cancer types. Even occupational exposure to acrylamide, resulting in acrylamide exposure nearly 10 times higher than dietary exposure, did not increase tumor occurrence. Furthermore, the consumption of coffee, which is a main contributor of dietary acrylamide exposure, actually decreases the overall incidence of cancer in humans and afford global health benefits, increasing both lifespan and healthspan on ageing. This paradox clearly illustrates the risk of evaluating an individual molecule independently of its complete food matrix, which may have other components that completely override the effects of the considered molecule.

## 1. Introduction

Acrylamide (AA), also known as 2-propenamide, is an organic compound that is soluble in water and organic solvents. AA is mostly found in cigarette smoke [1] and in a wide range of cooked and fried foods rich in carbohydrates, particularly potato chips, bread, crisps, breakfast cereals, and coffee [2,3]. AA can be formed in foodstuffs by the Maillard reaction (also known as food browning) during heat-induced processes, in which amino acids (mainly asparagine) react with reducing sugars (mainly glucose, fructose, maltose, and lactose) at temperatures above 120 °C [4].

As an α,β-unsaturated carbonyl with electrophilic properties, AA can react with biological nucleophilic groups of amines, carboxylates, aryl and alkyl hydroxyls, imidazoles, and thiols of macromolecules, especially cysteine residues, including DNA and proteins [5] via the Michael addition [6,7]. Thus, exposure to AA has the potential to affect a large number of biological constituents apart from the putative DNA reactivity of AA or of its cytochrome P450-metabolite, glycidamide, questioning the proposed main genotoxic mechanism of action of AA [8]. Concerns regarding the impact of AA on human health arose after the accidental exposure to AA-containing sealants in Swedish workers [9]. The carcinogenic potential of high doses of AA in rodents, alongside its presence in human foodstuffs and potential impact, led the International Agency for Research on Cancer (IARC) to classify AA as a probable carcinogen to humans (Group 2A; [10]).

However, the putative risk of AA to human health is critically dependent, amongst other factors, on the conditions of exposure, i.e., how AA is absorbed and is distributed in the human body as well as its excretion, and this potential toxicodynamic profile is affected by different other constituents present in the diet, such as garlic, blueberries, blackberries, grapes, strawberries, wolfberries, mulberries, wasabi, and green tea [11,12,13,14]. Furthermore, different constituents of any type of food may overcome and/or counteract any deleterious potential effects of AA on the human body. The aim of this review is to comparatively evaluate the scientific data on the health impacts of AA and coffee, which contains AA generated during green bean roasting, but also contains a variety of other bioactive molecules, including caffeine and numerous other substances, some with antioxidative potential. In fact, despite its AA content, coffee intake offers a paradoxical overall health benefit to adults and elderly people when consumed in moderate amounts.

## 2. Levels of Exposure to Acrylamide

AA toxicology was initially considered to be a hazard resulting from cumulative occupational or accidental exposure, in view of the widespread use of AA since the 1950s in flocculants suspended in irrigation and drinking water, soil conditioning, wastewater treatment, and the textile and paper industries (for review, see [15]). AA may also be present with polyacrylamide in different cosmetics and personal care products (such as lotions, deodorants, and shampoos), but in residual amounts that present little risk to human health [16,17]. AA is also found in cigarette smoke (1.1−2.3 µg per cigarette), constituting a non-negligible source of AA exposure [1,18]. However, the formation of AA in different foodstuffs as a result of heating when processed [19,20] generated large interest in the potential widespread exposure to AA (reviewed in [3,4,21,22,23]), wherein AA was found in potato chips (28–954 μg/kg), French fries (117–1325 μg/kg), biscuits (234−1104 μg/kg), coffee (210–960 μg/kg), white bread (42–231 μg/kg), breakfast cereals (41–726 μg/kg), baby food (<30–65 μg/kg), green and black olives (32–97 μg/kg), and nuts and dried fruits (2–93 μg/kg) (reviewed in [23,24]).

Based on dietary habits monitored in over 20 countries, European populations have a mean daily average intake of AA ranging from 0.14 to 1.31 μg/kg body weight (bw) [22]. Similar levels (0.43–1.1 μg/kg bw/day) were recorded in the USA [25]. Daily AA intake/kg bw may reach higher figures, especially in children who have larger relative intake based on body weight [22].

In the adult and elderly populations (20–79 years), the contribution of coffee to AA intake ranges from 9% to 29%, and reaches 38–60% for baked goods or crisps, depending on the country of origin, e.g., Canada, France, Poland, or Norway [22,25,26,27,28]. In a recent study, the consumption of four cups of coffee a day (total ingested AA dose of 7.9 μg = 0.15–0.17 μg/kg bw) was reported to result in AA levels just detectable by individual excretion kinetics, clearly indicating that normal coffee consumption would, in the worst case, contribute to overall dietary AA exposure to a minor degree [29]. Based on an average body weight of 70 kg, the daily intake of AA is estimated to range from 14 to 70 μg/day for adults considering data from various European, American, and Asian countries (see [27,30,31,32,33,34] as examples), implying that the relevance of any animal toxicological studies using excessively higher doses of AA to model human exposure to AA through food intake should be questioned.

Although acrylamide has known toxic effects on the nervous system and fertility, a 2002 FAO/WHO document concluded that the level of AA intake required to observe neuropathy (0.43 mg/kg bw/day) or effects on fertility was 500 or 2000 times higher than the average dietary intake (1 μg/kg bw/day), leading to the conclusion that AA levels in food are safe in terms of neuropathy, but raising concerns over human carcinogenicity, in spite of the high doses tested in rodents and the lack of clear epidemiological data in humans [35].

## 3. Animal Studies of AA Carcinogenicity

The carcinogenic potential of AA was taken into consideration mainly based on rodent studies after exposure to high doses of AA without conclusive support from epidemiological studies in humans, leading IARC to classify AA as a probable carcinogen to humans (Group 2A; [10]).

Two older, chronic carcinogenicity studies were carried out in rats, but used high daily doses corresponding to 20–5000 times the average exposure in humans [36,37]. The first study [36] revealed an AA-induced increase in the incidence of tumors of the mammary gland, central nervous system, thyroid gland follicular epithelium, oral tissues, uterus, and benign adenomas of other organs in female rats, and testicular mesothelium in male rats. The second study [37] only showed the emergence of tumors of the thyroid gland follicular epithelium in both males and females, scrotal mesothelium in males, mammary gland adenocarcinomas, and fibroadenomas in females.

In contrast, administration during 30 weeks of lower doses of AA, i.e., closer to those consumed by humans, was devoid of any overt effects on the health of rats [38]. Another careful study of increasing doses of AA in drinking water concluded that, after two years of continuous exposure, the highest average dosage, 0.70 mM, which was equivalent to about 2.7 mg/kg/day (males) and 4.0 mg/kg/day (females) and represents at least 3000 times the average dose consumed by humans, led to different types of neoplasms: in exposed rats, tumors were observed in lung (males and females), forestomach (males), mammary gland fibroadenomas, clitoral gland (papilloma and carcinoma), thyroid (males and females), ovary, and skin (females) [39].

The same study explored AA carcinogenicity in B6C3F1 mice; in these animals, with the exception of Harderian gland adenomas observed at lower doses, the occurrences of most other tumors (lung tumors in males and females, forestomach tumors in males, and mammary gland, ovary, and skin tumors in females) were only significantly increased at the two highest doses [39]. Older studies in other mouse strains receiving high doses of AA ranging from 12.5 to 50 mg/kg mostly reported AA-induced squamous cell adenomas and carcinomas in the skin of female Swiss mice [40], and adenomas and carcinomas in the lungs of female Swiss-ICR mice [41]. In addition, the consequences of AA exposure were shown to be not only species-specific but also strain-specific, since Wistar Han and Fisher 344 rats developed different types of tumors after AA exposure. These tumor developments in specific target tissues induced by high doses of AA in rodents indicate a lower likelihood of AA-induced cancer risk in humans than initial predictions [42,43,44,45,46].

In conclusion, it appears that long, continuous exposure to high doses of AA leads to species-specific and strain-specific effects in rodents, with the most sensitive neoplastic lesions occurring in the Harderian gland, which does not exist in humans. Moreover, most effects were observed after administration of AA in isolation and in excessively high doses (even considering different interspecies pharmacokinetic processing of AA) when compared to current human dietary exposure [47].

## 4. Impact of AA on Cancer Risk in Humans

The site most sensitive to AA-induced carcinogenesis is the Harderian gland, which is not present in humans. The other sites shown to be sensitive to high-dose AA exposure in rodents were thyroid, lung in both genders, ovary, skin, breast, clitoral gland, uterus in females, testicular mesothelium, and forestomach in males. Occasional AA-induced carcinogenesis was reported in the central nervous system and oral tissues in rodents [36,37,38,39,40,41].

Retrospective reanalyses of case-control studies of cancer incidence in European populations did not identify any causal relationship between consumption of foods or beverages containing AA and the incidence of cancers at various sites [48]. Furthermore, reviews and meta-analyses performed in humans did not report any association between dietary AA intake and the risk of cancer for most organs susceptible to AA-induced carcinogenesis in rodents. This was shown to be the case for lung [49], breast cancer [50,51,52,53,54], and most studies of ovary cancer [52,55,56]. Only one recent study [54] reported a slight increased risk of ovarian cancer upon AA exposure. In humans, the data on AA and endometrial cancer risk are somewhat inconsistent. While some studies and meta-analyses reported no association between dietary AA intake and endometrial cancer [52,57,58], others reported increased risk [51,54,56]. No associations were reported between AA intake and the risk of skin cancer, particularly in females, which represented the most sensitive gender for this type of cancer in rodents [59]. Repeated studies of workers exposed to AA revealed no cases of brain tumors in exposed workers (for review, see [15]), and dietary AA was not associated with brain cancer risk [60]. Systematic reviews, meta-analyses, and large prospective studies did not report any association between AA and the risk of stomach cancer [58,61] or the risk of head-and-neck [61,62], oral, or pharyngeal cancers [58], including thyroid cancer [63], although in this latter case, a few cases were reported in female nonsmokers [63].

Concerning the sites not shown to be sensitive to AA-induced cancer in animals, AA is not associated with any digestive, esophageal, pancreatic, or colorectal cancers in humans [58,61,64]. No association was found between dietary AA and risk of total or different grades of prostate [58,65] or bladder cancer [48,64,66]. Two long-term, recent, prospective cohorts reported no association between AA intake and the risk of renal carcinoma in either gender [67], while earlier studies found borderline positive associations [58,64]. Additional meta-analyses suggested no association between AA and lymphomas [58] (also see [68].)

## 5. Impact of Coffee on Cancer Risk in Humans

The current uncertainty around the carcinogenic potential of AA consumed through a regular diet is best heralded by the comparison of the purported effects of AA with those associated with the intake of coffee, which is often argued to be a main AA-containing foodstuff [22,25,26,27,28], considered to represent 9–29% of daily nutritional AA intake [22].

Most reviews on the consequences of lifelong coffee intake on cancer concluded that coffee consumption does not increase the risk of developing cancer in most sites and even reduces the risk of cancer in some locations, such as liver, ovary, thyroid, endometrium, gastrointestinal tract, and skin melanoma. There is no causal relationship between coffee and risk of breast, lung, bladder, pancreas, or prostate cancer, as repeatedly reported in reviews and meta-analyses [69,70,71,72,73,74,75,76]. In 2016, a panel of experts from the International Agency for Research on Cancer (IARC) Working Group re-evaluated the effect of coffee intake on cancer incidence. After reviewing more than 1000 studies in humans and animals, the Working Group concluded that there was no evidence supporting the carcinogenicity of coffee drinking overall, and classified coffee in Group 3, i.e., “not classifiable as to its carcinogenicity to humans” [77,78].

Since the IARC report in 2016 [77], a number of meta-analyses and prospective studies reported either reduced risk or no influence of coffee on the risk of cancers at sites previously reported in rats and mice to be vulnerable to AA at doses exceedingly higher than regular human exposure to AA via food and drink [36,37,38,39,40,41]. Thus, two meta-analyses found no association between coffee and thyroid cancer risk [79,80], and a more recent one even found an overall decreased risk of thyroid cancer of up to 25%, and a 5% decrease for each additional cup of coffee [81]. In parallel, recent meta-analyses and large studies showed no association between coffee consumption and lung cancer, as long as the potential confounding effect related to smoking is accounted for [82,83,84,85].

Most studies and meta-analyses found no relationship between coffee intake and ovarian cancer [86,87,88]. Interestingly, one meta-analysis reported even an inverse association between coffee drinking and ovarian cancer, but only for decaffeinated coffee, with no relationship in regard to regular coffee [88]. On the same note, most studies found no association or even a slight risk reduction between coffee consumption and breast cancer in general [89,90]. In subgroups, coffee intake decreased risk (up to 24–44%) in postmenopausal women [89,90,91], estrogen-receptor-negative breast cancer [92], and possibly in female carriers of the BRCA1 and BRCA2 mutations [89,93].

Interestingly, coffee consumption was found to consistently reduce the risk of developing endometrial cancers by about 20% in both cohort and case-control studies [90,94,95,96]; this is one of the most robust and reproducible effects of coffee consumption on cancer, as highlighted by IARC (2018). A recent meta-analysis with a dose-response analysis, including 12 cohort and 8 case-control studies, strengthened the evidence of a protective effect of coffee consumption on the risk of endometrial cancer, and further suggested that increased coffee intake might be particularly beneficial for women with obesity [97].

Concerning skin, an organ often found as a target for AA-induced carcinogenesis in animal studies [36,37], meta-analyses indicate borderline protective effects of coffee consumption against melanoma [98,99,100] and basal cell skin cancers [101,102,103]. On a similar note, no association was found between coffee consumption and the risk of brain tumors [104,105] or gliomas [106,107,108], as reported in meta-analyses and reviews. Similarly, no association was found between coffee consumption and oral or pharyngeal cancer risk [109,110]. Also, meta-analyses report no association between coffee intake and stomach cancer [111,112], with two studies even reporting decreased risk [75,113]. Only one study reported increased risk in the USA, but only for a relatively high consumption, i.e., 6.5 cups/day [114].

The consumption of coffee was concluded to be highly protective against liver cancer and most liver diseases [115,116]. Coffee intake displayed a robust and consistent risk reduction of hepatocellular carcinoma of 35–50%, as reported in cohort studies, case-control studies, reviews, and meta-analyses [115,117,118,119]. A 15% decreased risk in liver cancer was observed for an increment of one cup of coffee per day [117,120], making this reduction of liver carcinoma the most prominent protective effect afforded by coffee consumption. Besides this effect on liver carcinoma, coffee intake also decreases the occurrence of nonalcoholic fatty liver disease (NAFLD [121]), and is beneficial in hepatitis B and C [122].

In colorectal adenoma, coffee drinking is borderline protective [123,124,125]. For pancreas cancer, there is no association between coffee exposure and cancer risk [77,78,126,127,128,129]. Some concern was raised about a possible link between coffee consumption and esophageal cancer in older studies, linked to the earlier observation that mate and hot tea drinking were associated with esophageal cancer [77,78]. However, the increase in esophageal cancer risk relates to the elevated temperature, over 60 °C, at which mate and tea are often consumed [130]. Two meta-analyses reported that when coffee is consumed at regular temperatures, lower than 60 °C, it is not associated with esophageal cancer [131,132].

The IARC experts also concluded a lack of association between coffee intake and prostate cancer risk [77,78]. More recent studies confirm this conclusion, with no evidence of association found regarding the consumption of total, caffeinated, or decaffeinated coffee and risk of total prostate cancer or cancer by stage, grade, or fatality in the large European Prospective Investigation into Cancer and Nutrition (EPIC) cohort [133,134].

A cohort study and a meta-analysis found no association between coffee drinking and bladder cancer risk [72], which was confirmed in a recent meta-analysis of 16 prospective studies [135]. Likewise, meta-analyses suggest no association or a slightly reduced risk of kidney cancer in coffee drinkers [136,137]. Concerning lymphoma, meta-analyses suggest no association with coffee intake [138,139].

In conclusion, the exposure to AA from food sources does not appear to increase the risk of cancer in humans, even in locations prone to developing cancer in rodents exposed to relatively high doses of AA. In addition, no evidence exists that the daily consumption of coffee, which contains AA (9–29% of daily AA intake, [22]), would be associated with an increased risk of cancer at any location. On the contrary, at some sites, mainly the liver and endometrium, there are strong inverse associations between coffee intake and cancer risk. Indeed, the American Cancer Society concluded in their guidelines that coffee reduces the risk of skin, mouth, and throat cancer, and likely also reduces the risk of liver and endometrial cancer [140]. At most other sites reviewed, the IARC group of experts considered that there is no evidence in humans regarding a carcinogenicity upon coffee exposure [77,78], as confirmed by more recent reviews and meta-analyses. Hence, it appears that there is no potential carcinogenic role of coffee or AA ingestion via coffee. This is either because AA is not carcinogenic per se at the doses ingested through the diet, and/or because coffee possesses other constituents with beneficial effects on human health, thereby overriding any potential detrimental effect of AA. However, although coffee intake and the estimated contribution of coffee intake to total AA dietary exposure are considered in most epidemiological studies, with some notable exceptions where an interaction between both factors was evaluated with negative results [51,52,68], it is not clear if the lack of effects of AA intake on cancer risk are associated with a protective effect of coffee. This was hinted at in in vitro studies showing that a prominent coffee constituent—caffeine—counteracts the impact of AA on cellular arrest and apoptosis [141,142]. The anticancer properties of coffee compounds, most often based on animal in vitro or ex vivo studies, were recently reviewed [143], showing that different coffee constituents present in measurable or high concentrations act along different pathways of carcinogenesis. In addition to caffeine, other coffee constituents, namely antioxidants such as phenolic acids (chlorogenic, caffeic, and ferulic acids), melanoidins, and the diterpenes kahweol and cafestol, are involved at various individual steps critical for the survival of cancer cells [144,145,146,147,148,149]. This provides a biological rationale sustaining the potential protection afforded by coffee intake against the development of some types of cancer.

## 6. Other General Beneficial Health Effects of Coffee

In addition to the properties of coffee in decreasing the risk of some cancers, the consumption of coffee has protective effects in several diseases, including neurodegenerative diseases, cardiovascular function, type 2 diabetes, depression and suicide risk, and even mortality risk.

Probably the most striking effect of coffee intake is the ability to increase lifespan and, most importantly, healthspan on ageing [106,150,151,152,153,154,155,156,157], as concluded from the analysis of different cohorts in Europe [158,159,160,161,162,163,164], America [165,166,167], and Asia [168,169,170,171], with different ethnicities [170] and different types of coffee [167]; these effects were observed similarly in both men and women [152,161] with different polymorphisms [162,172], sharply contrasting with the positive association of life-long acrylamide exposure [173] and acrylamide–hemoglobin adducts [174] with mortality.

This positive association between the regular intake of moderate doses of coffee and the increased longevity and health quality with ageing is tightly related to the ability of coffee intake to decrease the risk of developing major age-releated chronic disorders, such as diabetes [175,176,177,178,179,180], cardiovascular diseases [150,181,182,183,184,185,186,187,188], stroke [189,190,191,192], depression and suicide [193,194,195,196,197,198,199,200,201], cognitive decline [202,203,204,205,206,207], and neurodegenerativce diseases, such as Alzheimer’s disease [208,209,210,211,212] and Parkinson’s disease [213,214,215,216,217,218].

## 7. Conclusions

The present review reports that, in addition to the anticarcinogenic effects of coffee observed despite the presence of variable amounts of AA in the beverage due to the roasting process, coffee consumption also exerts positive effects on health and well-being and prevents various pathologies throughout many central and peripheral organs.

Three reviews on the consequences of coffee consumption on health were recently published. The first one reviewed data of 112 meta-analyses for 59 unique health outcomes and concluded the following: “Given the spectrum of conditions studied and the robustness of many of the results, these findings indicate that coffee can be part of a healthful diet” [219]. The second reviewed data from 201 meta-analyses with 67 unique health outcomes and 17 meta-analyses of interventional studies with nine unique outcomes, concluding: “Coffee consumption seems generally safe within usual levels of intake, with summary estimates indicating largest risk reduction for various health outcomes at three to four cups a day, and more likely to benefit health than harm” [220]. The third one [221], including 95 studies, concluded the following: “In fact, consumption of 3 to 5 standard cups of coffee daily has been consistently associated with a reduced risk of several chronic diseases. … Current evidence does not warrant recommending caffeine or coffee intake for disease prevention but suggests that for adults who are not pregnant or lactating and do not have specific health conditions, moderate consumption of coffee or tea can be part of a healthy lifestyle”.

In our opinion, there is enough evidence available in the literature on coffee consumption and health to conclude that coffee has no harmful effects, but instead is overall benefitial on human health, be it cancer or other critical health outcomes. These positive outcomes occur in spite of the presence of AA in coffee, which most likely reflect the powerful effects of all other components of coffee with allostatic, anti-inflammatory, antioxidant, and radical scavenging properties, which are also able to normalize body functions, limit DNA damage, and stimulate the activity of detoxifying enzymes. The perception that the AA levels present in coffee might have some negative impact on human health was previously a major driving force to develop several strategies to decrease the levels of AA in coffee (reviewed in [23,222]). However, such an aim might actually be more harmful than beneficial if these mitigation strategies also interfere with other compounds present in coffee that may be responsible for the health benefits of coffee, again stressing the problems of considering one particular component independently of its food matrix. It is also clear that the analysis of coffee consumption on health requires a holistic approach, and the analysis of the health consequences of ingestion of the whole drink, in which AA is present together with protective molecules, and not an isolated, residual, harmful component.
